# Comprehensive analysis of mRNA-lncRNA co-expression profile revealing crucial role of imprinted gene cluster DLK1-MEG3 in chordoma

**DOI:** 10.18632/oncotarget.22616

**Published:** 2017-11-08

**Authors:** Hao Chen, Kai Zhang, Jian Lu, Guizhong Wu, Huilin Yang, Kangwu Chen

**Affiliations:** ^1^ Department of Orthopedics, The First Affiliated Hospital of Soochow University, Suzhou, Jiangsu 215006, P.R. China; ^2^ Institute of Orthopedics, Soochow University, Suzhou, Jiangsu 215006, P.R. China

**Keywords:** chordoma, long non-coding RNA, protein coding gene, imprinted gene cluster, DLK1-MEG3

## Abstract

Chordoma is a rare bone tumor with high recurrence rate, but the mechanism of its development is unclear. Long non-coding RNAs(lncRNAs) are recently revealed to be regulators in a variety of biological processed by targeting on mRNA transcription. Their expression profile and function in chordoma have not been investigated yet. In this study, we firstly performed the comprehensive analysis of the lncRNA and coding genes expression analysis with three chordoma samples and three fetal nucleus pulposus tissues. lncRNA and gene microarrays were used to determine the differentially expressed lncRNAs and protein coding genes. 2786 lncRNAs and 3286 coding genes were significantly up-regulated in chordoma, while 2042 lncRNAs and 1006 coding genes were down-regulated. Pearson correlation analysis was conducted to correlate differentially expressed lncRNAs with protein coding genes, indicating a comprehensive lncRNA-coding gene co-expression network in chordoma. Cis-correlation analysis showed that various transcripts of MEG3 and MEG8 were paired with the most differentially expressed gene DLK1. As located in the same locus, we further analyzed the miRNA clusters in this region, and identified that 61.22% of these miRNAs were significantly down-regulated, implying the silence of the imprinted gene cluster DLK1-MEG3. Overexpression of MEG3 suppressed the proliferation of chordoma cells. Our study pointed out the potential role of lncRNAs in chordoma, presented the lncRNA-coding genes co-expression profile, and revealed that imprinted gene cluster DLK1-MEG3 contributes to the pathogenesis of chordoma development.

## INTRODUCTION

Chordoma is a rare bone tumor with an incidence rate of 0.08/100000. It is widely acknowledged that chordoma is arisen from the remnants of the notochord, with the most common site being the sacrum, skull base and mobile spine [1]. Chordomas are histologically classified as low-grade neoplasm, whereas, its high recurrence rate makes this tumor very similar to malignant neoplasms [2, 3]. Currently, the available management for controlling chordoma includes surgery followed by radiation therapy, which is thought to be successful. However, the tendency to recur and metastasize makes this treatment still in a challenge [4]. Recent studies indicated that the growth of chordoma is possibly relying on multiple mechanisms, including genetic and epigenetic modifications. However, the precise mechanisms that promote the development of chordoma remain poorly understood [5, 6]. Epigenetic is the study to understand the various mechanisms that result in mitotically heritable changes in gene expression that do not involve any changes in DNA sequences [7–9]. The major epigenetic changes include DNA methylation, histone modification and post-transcriptional regulation controlled by non-coding RNAs. Based on previous studies, DNA hypermethlation and histone deacetylases were found in chordoma tissues, suggesting aberrant epigenetic modifications may attend in the development of chordoma [6].

Highly expressed non-coding RNAs compose most of the cellular RNA, and their relevance in cell functionality has been known for years. In chordoma, non-coding RNAs have been demonstrated to participate in various biological processes. For instance, microRNAs (miRNAs), one of the subtypes of non-coding RNAs, were found to be ectopically expressed in chordoma samples and cell lines. miRNA-1, one of the miRNAs, was observed to significantly under express in chordoma[10, 11], and re-expression of miRNA-1 in chordoma cell line decreased cell growth and proliferation by targeting on MET[12]. Besides, recent findings suggest that another subgroup of non-coding RNAs, which have the transcripts longer than 200 nucleotides in length, are also observed to abnormally express in human cancers [13–15]. Accumulating evidence demonstrated that these long noncoding RNAs (lncRNAs) are able to regulate cancer development, metastasis and chemo/radio-resistance by governing a wide repertoire of molecular biological and genetic processes, including transcription, translation, splicing and chromatin modification, etc [16–22]. lncRNAs can be classified as *cis* and *trans* acting molecules. *Cis*-acting lncRNAs function at the site of transcription and influent their neighbouring genes expression. *Trans*-acting lncRNAs function away from the site where it synthesized. Several *cis*-acting lncRNAs are able to guide epigenetic regulator to the transcription site, in order to create an anchor to recruit chromatin remodeling related proteins [23–26]. *Cis*-acting lncRNAs are found to control the epigenetic regulation of some imprinted genes. Imprinting depends on the parental origin of the genes, which play crucial roles in development [27]. The expression of imprinted genes must be tightly controlled, and many imprinted gene locus express lncRNAs that were found to regulate the neighbouring imprinted protein-coding genes expression in *cis*, allele specifically [28]. For example, the lncRNA *AIR* can silence the nerighbouring imprinting genes including *SLC22A3* and *IGF2R* [29].

In the current study, we performed microarrays to detect the lncRNAs and protein coding genes expression profile of chordoma and the control tissue fetal nucleus pulposus (FNP), in order to find out the lncRNAs involved in chordoma biological processes. In addition, we constructed the co-expression network of lncRNA-coding gene to predict the potential function of the differentially expressed lncRNAs. Among all the differentially expressed coding genes and lncRNAs screened, we identified that the imprinted gene cluster in the DLK1-MEG3 locus might contribute greatly to chordoma development. Our study could be useful to better understand the interplay between non-coding RNAs and coding gene transcripts in chordoma biology.

## RESULTS

### Demographic and clinical characteristics of three sacral chordoma

**Figure 1:**
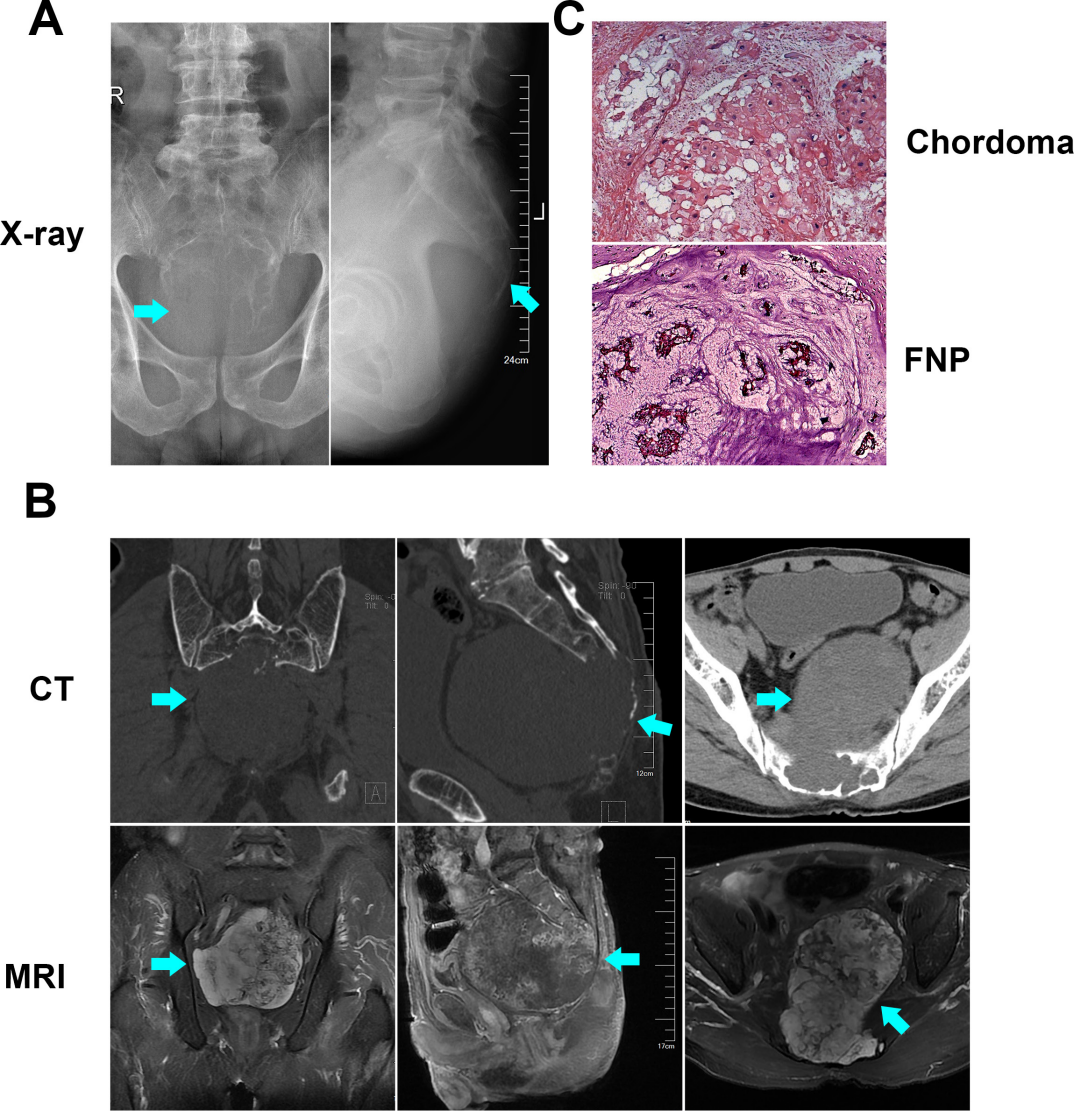
Radiological and pathological identification of chordoma X-ray film (**A**), CT and MRI images (**B**) showed the radiological presentation of sacral chordoma. HE staining (**C**) showed the histomorphology of chordoma and FNP samples that we collected.

Three patients diagnosed with primary sacral chordoma under tumor resection were enrolled in our study. The images, including X-ray, computed tomography (CT) and magnetic resonance imaging (MRI), showed typical image manifestation of chordoma in the sacrum. On the X-ray (Figure 1A) and CT images (Figure 1B), large lytic tumor lesion was observed in the midline and an associated soft-tissue mass, and sacrum bone destruction could be seen. Based on MRI (Figure 1B), the tumor lesion showed hyper-intense on T2 weighted images. HE staining (Figure 1C) and immunohistochemical staining (Cytokeratin and S100, data not shown) further indicated that all the three samples collected were chordomas belong to the classic category. The results confirmed that the tissue we harvested were classic chordomas.

### Overview of the protein-coding genes and lncRNA expression profile in chordoma and FNP tissue

**Figure 2:**
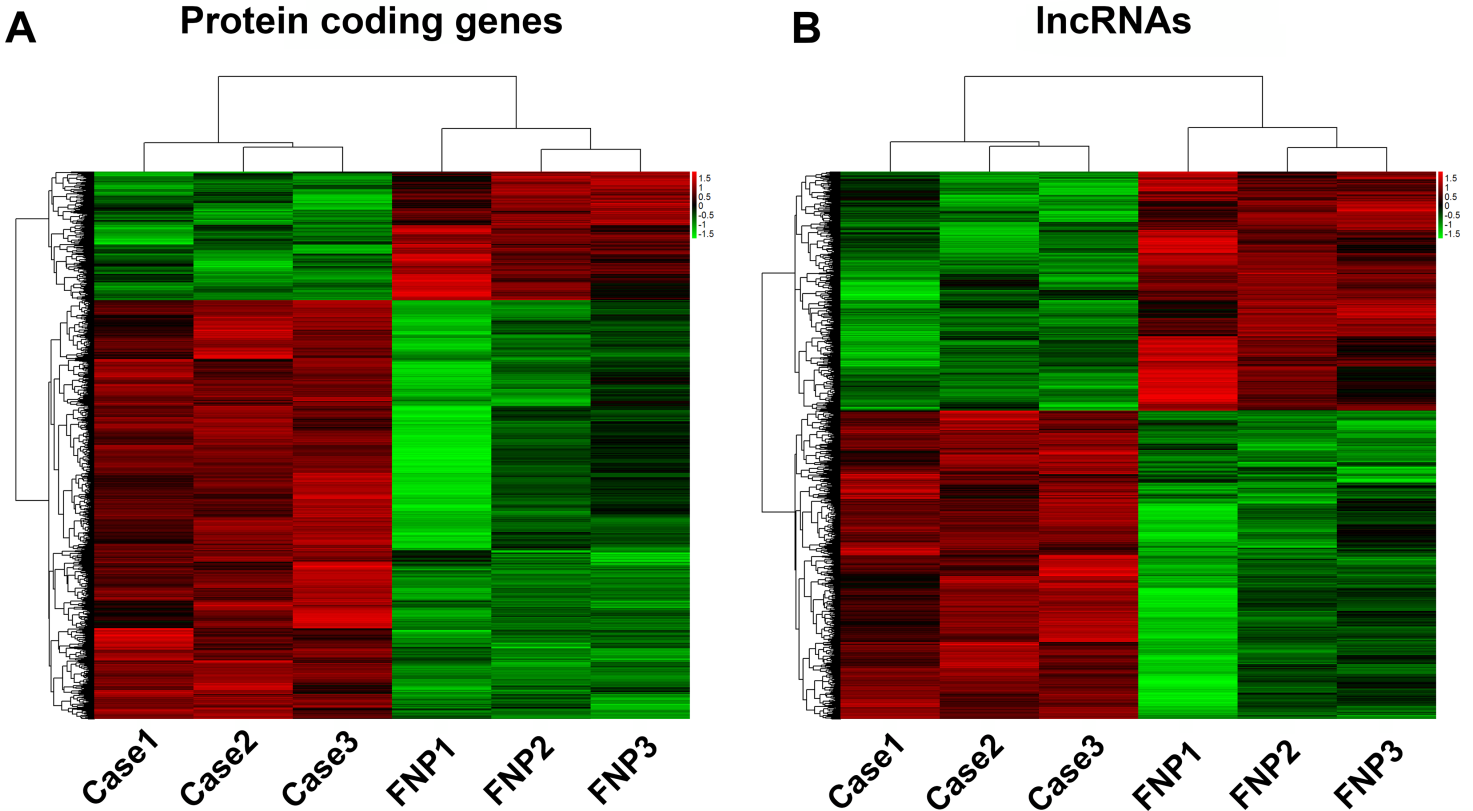
Heat maps of differential expression and hierarchical clustering of (**A**). Protein coding genes and (**B**). lncRNAs in chordoma and FNP tissues.

In order to determine differentially expressed protein-coding genes and lncRNAs, we performed microarray analysis on chordoma and fetal nucleus pulposus tissue. Based on the expression level of all the RNAs screened in the microarray, hierarchical clustering analysis was performed to group protein-coding genes and lncRNAs. The results suggested that the samples come from the same cluster within the case or control group (Figure 2).

**Figure 3:**
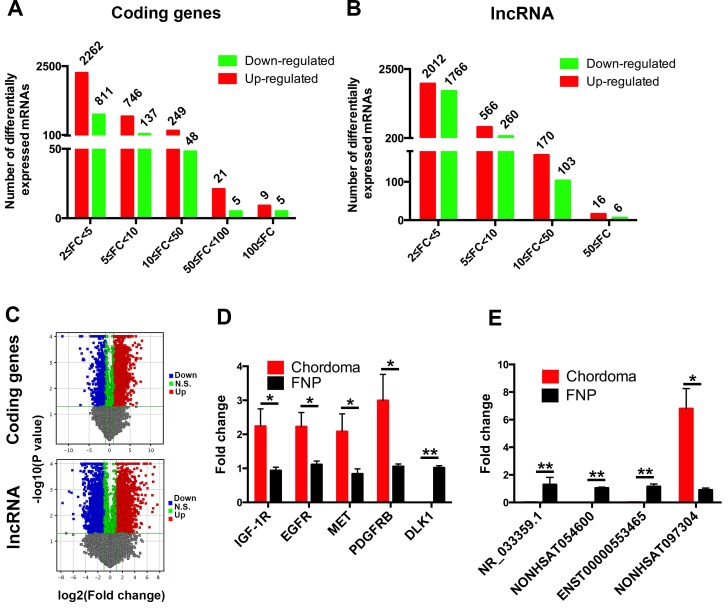
Profile and verification of the differentially expressed mRNAs and lncRNAs Microarray analysis of the chordoma samples and FNP revealed the number of differentially expressed protein coding genes (**A**) and lncRNAs (**B**) with various fold changes. The volcano plots (**C**) showed the distribution of differentially expressed protein coding genes and lncRNAs identified in chordoma. The protein coding genes or lncRNAs with more than 2 fold change were noted as red dots(positive) and green dots(negative). Randomly selected protein coding genes (**D**) and lncRNAs (**E**) were validated by qPCR. (^*^ represents for *P* value < 0.05, and ^**^ represents for *P* value < 0.01).

Differentially expressed protein-coding genes and lncRNAs were selected when they were altered by fold change no less than 2.0 (*P* value < 0.05). A total of 4292 protein-coding genes and 4899 lncRNAs were found to differentially express in chordoma. Concerning to the protein-coding genes expression with more than 100 fold change, there were 9 up-regulated (HLA-DQA1, HLA-DPB1 and C1QB, etc.) and 5 down-regulated genes (DLK1, UCMA and LECT1, etc.). Most of the differentially expressed genes belonged to the 2 ≤ FC < 5 group (Figure 3A). Similar to the differentially expressed protein-coding genes, 16 up-regulated lncRNAs (NONHSAT060785, NONHSAT116806 and NONHSAT104550, etc.) and 6 down-regulated lncRNAs (NONHSAT113429, NONHSAT113428 and NONHSAG044080, etc.) were identified with expression change more than 100 fold, and the group with most differentially expressed lncRNAs was 2 ≤ FC < 5 (Figure 2B). The list of differentially expressed protein-coding genes and lncRNAs were shown in Supplementary Table 1 and Supplementary Table 2. To further exhibit the distribution of these differentially expressed protein-coding genes and lncRNAs, we constructed the volcano plots. As shown in Figure 3C, The significance versus fold change was plotted into volcano plots, which reflected the distribution of the screened protein coding genes and lncRNAs between chordoma and FNP samples.

To validate the microarray results, we selected 5 coding genes and 4 lncRNAs for quantitative polymerase chain reaction (qPCR). The results showed that IGF-1R, EGFR, MET and PDGFRB expression was significantly higher in chodoma, and DLK1 expression was decreased as shown in microarray data (Figure 3D). qPCR analysis of the randomly selected lncRNAs revealed that NR_033359.1, NONHSAT054600 and ENST00000553465 were significantly lower in chordoma, and NONHSAT097304 expression was much higher in chordoma as reflected by microarray analysis (Figure 3E).

### Subgroups classification of differentially expressed lncRNAs

**Figure 4:**
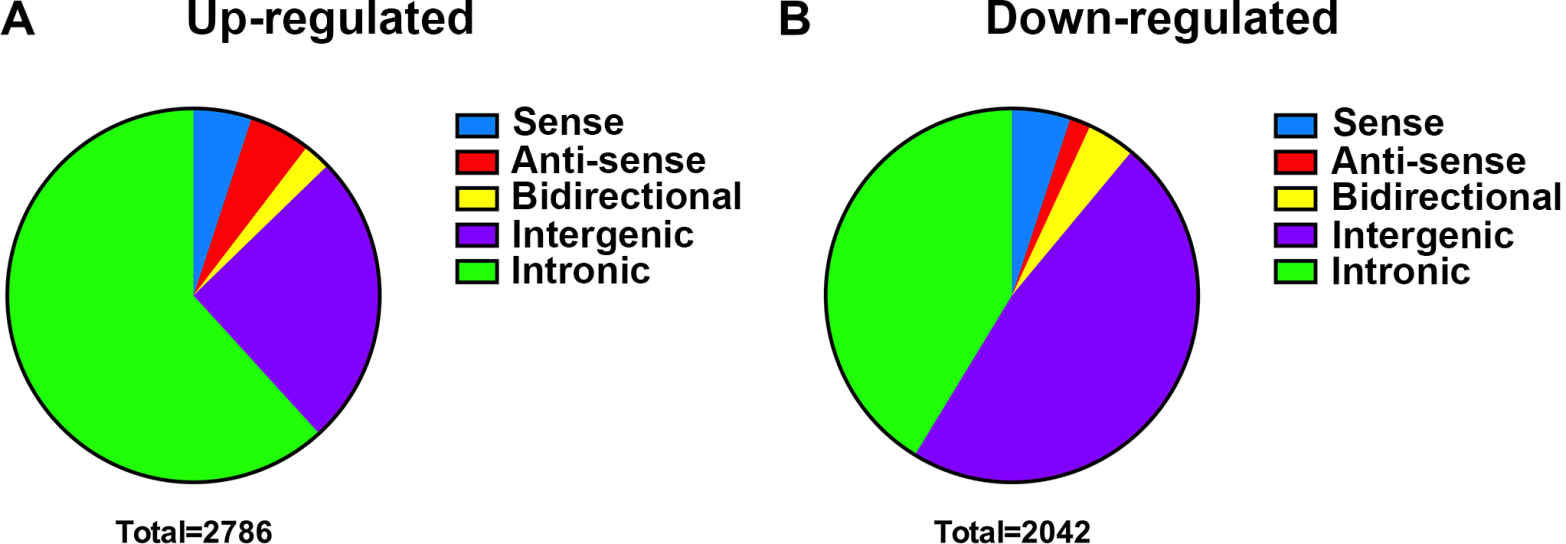
Subgroups classification of differentially expressed lncRNAs The subgroups of differentially expressed lncRNAs were classified as sense, anti-sense, bidirectional, intergenic and intronic. The up-regulated lncRNAs (**A**) and down-regulated lncRNAs (**B**) were classified into the five groups.

We continuously adapted specific probes for lncRNAs to further classify the differentially expressed lncRNAs in accordance with their different features. As shown in Figure 4, there were 141 up-regulated and 104 down-regulated sense lncRNAs, and 149 up-regulated and 85 down-regulated anti-sense lncRNAs were found as well. The profile data suggested 66 bidirectional lincRNAs were up-regulated, and 37 were down-regulated. As for intergenic lncRNAs, 709 were up-regulated and 973 were down-regulated. Meanwhile, we also identified 1721 up-regulated and 843 down-regulated intronic lncRNAs in chordoma.

### Construction of the lncRNAss and protein-coding genes co-expression network

**Figure 5:**
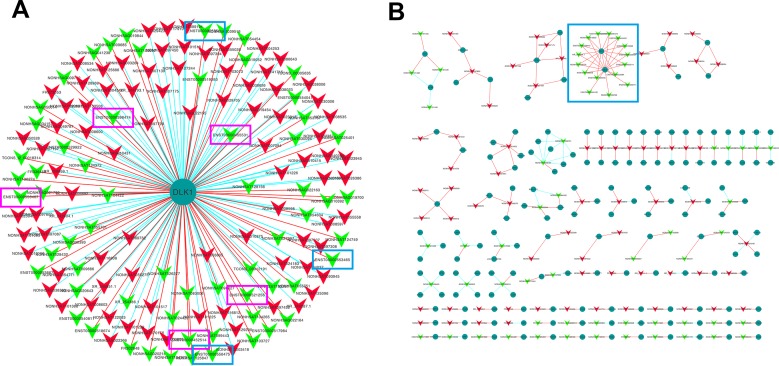
Construction of the lncRNAs and protein coding genes co-expression network (**A**) The differentially expressed lncRNAs that correlate with DLK1. The red triangle represents the up-regulated lncRNAs and the green triangle represents the down-regulated lncRNAs. Five transcripts of MEG3(pink rectangles) and three transcripts of MEG8(blue rectangles) that correlate with DLK1 were labeled. (**B**) *Cis*-regulation analysis of the lncRNAs(red and green triangles) that correlate with protein coding genes(deep green circles). MEG3 and MEG8 were identified to be *cis*-correlated with DLK1(blue rectangle). Red line represents positive correlation, and blue line represents negative correlation.

To further explore the potential regulatory mechanism of the differentially expressed lncRNAs, the lncRNAs and protein-coding genes co-expression network was constructed based on correlation analysis. Pearson correlation coefficient analysis was performed to evaluate the correlation between lncRNAs and coding genes, and the lncRNAs and coding genes with Pearson correlation coefficient no less than 0.90 and the *P* value less than 0.01 were picked up for network construction. The results of lncRNA and coding genes correlation analysis were listed in Supplementary Table 3. As DLK1 was found to be the most differentially expressed protein-coding gene in chordoma (down regulated with FC 2771.79), we analyzed the lncRNAs that correlate with DLK1 specifically (Figure 5A). Interestingly, we found that 5 transcripts of maternal expressed gene 3 (MEG3) and 3 transcripts of MEG8 were correlated with DLK1 (Figure 5A), indicating the possible regulatory role of MEG3 and MEG8 for DLK1.

### *Cis*-regulation of lncRNAs

As lncRNAs are physically linked to the locus from which they are encoded, they may have the ability to function during or immediately following the transcription process. To understand the *cis*-regulation of lncRNAs in chordoma, the lncRNAs and the associated protein coding genes that screened by Pearson correlation coefficient (Correlation ≥ 0.90. *q* value < 0.01) were further mapped to their genomic locus. lncRNAs which locate within 300K windows upstream or downstream of the given protein coding genes were further analyzed. The most 200 over or under expressed lncRNAs were selected in this analysis, in which, 129 were located. The lncRNA-coding gene pairs for *cis*-regulation were shown in Figure 5B. We noticed that several of the down-regulated lncRNAs were all targeting on DLK1, the most under expressed coding gene in chordoma. There are several isoforms of MEG3 and MEG8, among which, five of them were thought to *cis*-correlate with DKL1, including ENST00000398474 (MEG3-005), ENST00000455531 (MEG3-008), ENST00000452514 (MEG3-023), ENST00000521256 (MEG3-024), ENST00 000556407 (MEG3-025), ENST00000556475 (MEG8- 006), ENST00000553584 (MEG8-015) and ENST00000 553465 (MEG8-016) (Figure 5B). Concomitant down-regulation of the DLK1 and MEGs imply that imprinted genes may contribute to the development of chordoma.

### Concomitant down-regulation of the imprinted genes DLK1 and MEG3 potentially silenced the corresponding miRNA clusters expression

**Figure 6:**
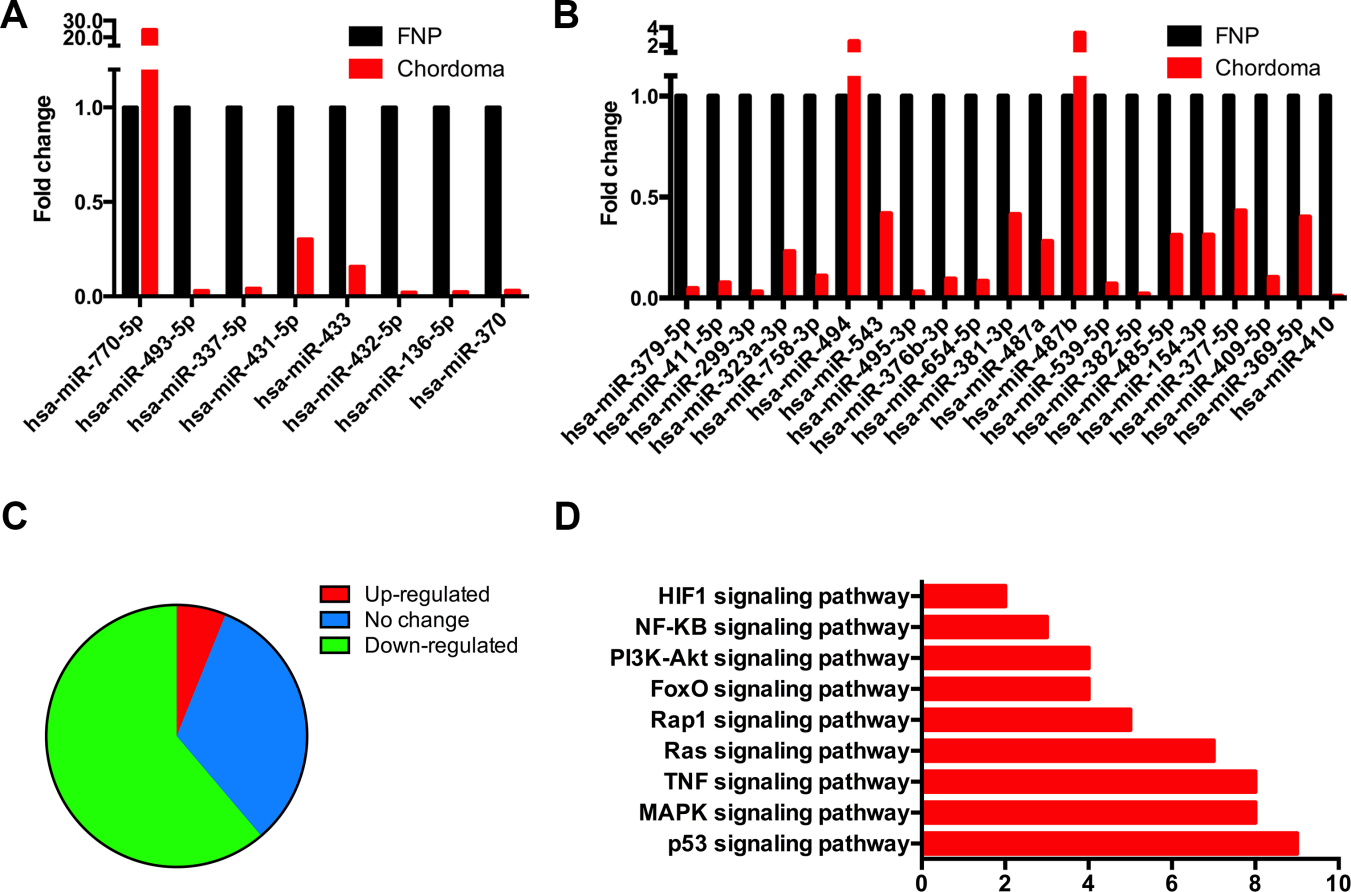
Suppressed expression of the imprinted gene clusters in DLK1-MEG3 region The chordoma samples were subjected to miRNA microarray analysis. (**A**, **B**) Expression of the two major miRNA clusters in DLK1-MEG3 region in chordoma. (**C**) Percentage of the up-(red) and down-(green) regulated miRNAs in the DLK-MEG3 region in chordoma. (**D**) KEGG analysis of the genes that correlate with MEG3 and MEG8 were enriched in different signaling pathways.

As reported, a cluster of imprinted genes at 14q32.2, the DLK1-MEG3 locus, were found silence in several types of cancers [30], and loss of imprinting genes impairs various biological processes [31]. Our lncRNA-coding genes profile data showed that the most differentially expressed protein coding gene in chordoma was DLK1, and *Cis*-regulation analysis revealed that this gene was targeted by a bunch of lncRNAs, most of which were identified to be different transcripts of MEG3 and MEG8. They were concomitantly down-regulated in chordoma. As miRNAs cluster consists the major part of the DLK1-MEGs locus, and DLK1-MEG3 silencing induced miRNA suppression were found to appear in a few pathological processes [32]. Therefore, we performed miRNA microarray analysis on the chordoma samples used in the lncRNA-coding gene expression analysis. The clusters of miRNAs that locate in the 14q32.2 locus were analyzed, for which, the miRNAs expression with no less than two-fold change were considered as different. The relative expression value of the miRNAs clusters in the DLK1-MEG3 locus was presented in Figure 6A (miRNA cluster A) and 6B (miRNA cluster B), showing that most of the miRNAs were down-regulated. Totally, 30 (61.22%) miRNAs were identified to be down-regulated, and 3 (6.12%) miRNAs were up-regulated in the cluster of 14q32.2 in chordoma, and 16 miRNAs remain unchanged (Figure 6C). Furthermore, we did KEGG analysis of the genes that correlated with MEG3 and MEG8, revealing that these genes were enriched in p53, MAPK and TNF et al. signaling pathways (Figure 6D), most of which are active in cancer biology. The results above validated that DLK1-MGE3 locus was silenced in chordoma, and it might contribute to the development of this neoplasm.

### Over-expression of MEG3 suppresses chordoma cells proliferation

**Figure 7:**
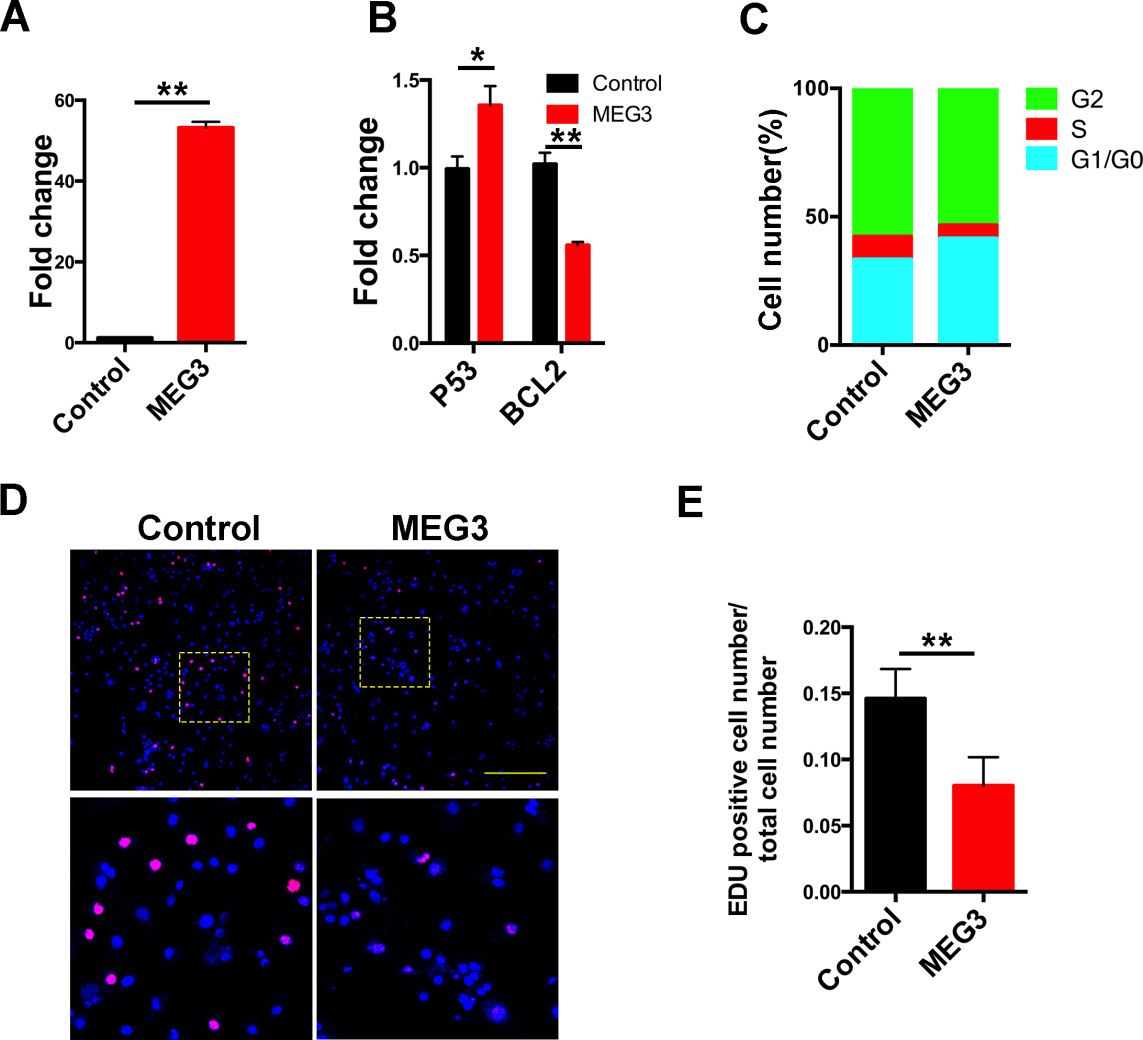
Over-expression of MEG3 suppressed chordoma cells proliferation The chordoma cells were transfected with pcDNA-MEG3 to elevate MEG3 expression in chordoma cells. qPCR validated that MEG3 expression is significantly up-regulated in chordoma cells (**A**), and its down-stream targets P53 and Bcl2 were also changed (**B**, **C)**. Cell cycle analysis showed the chordoma cells were retained in the G1/G0 phase with MEG3 over-expression. (**D**, **E**) EdU staining of the chordoma cells showed the decreased proliferation rate in MEG3 over-expression group.(^*^ represents for *P* value < 0.05, and ^**^ represents for *P* value < 0.01).

As determined by qPCR, MEG3 expression increased significantly in chordoma cells after transfection with the pcDNA-MEG3 (Figure 7A). The expression of its down stream target genes including P53 and Bcl2 was also changed significantly (Figure 7B), indicating successful over-expression of MEG3 in chordoma cells. Increased P53 and decreased Bcl2 expression may also imply the enhanced apoptosis in chordoma cells with MEG3 over-expression. Cell cycle analysis revealed that MEG3 increased the percentage of cells in the G1/G0 phase compared with control (Figure 7C). Meanwhile, EdU staining further demonstrated that the proliferation rate was significantly lowered in MEG3 over-expression group comparing with the control group (Figure 7D and 7E). The results suggest that MEG3 can effectively suppress chordoma cells proliferation.

## DISCUSSION

Chordoma is a rare bone tumor with the characterization of chemo- and radio-resistance. The extremely high recurrence rate of chordoma poses this tumor on the borderline of low-grade and malignant neoplasm [1]. Although chordoma has been characterized for more than a century, little of the fundamental mechanisms and structures in the initiation and progression of chordoma is known. Growing evidence suggests the emerging role of lncRNAs in the various biological process influencing cellular metabolism and development [33]. The newly characterized non-coding RNAs have drawn the scientific attention since their indispensable roles in cancer development and progression [34]. Benefitting from the advance of new technology achieving unprecedented depths in RNA sequencing, a large pool of lncRNAs have been identified to spread over the mammalian genome. As the integration analysis of lncRNA-coding gene is one of the most common approaches to predict lncRNAs functions in different biological processes, therefore, we comprehensively analyzed the lncRNAs and mRNAs in order to predict their potential functions in chordoma.

Our micro-array data revealed that a large number of genes were differentially expressed in chordoma tissues, including EGFR [35, 36], PDGFRβ [37], IGF-1R [38, 39] and MET [40, 41] etc., which have been identified to the potential therapeutic targets in chordoma with both clinical and molecular evidence. Unlike gene coding RNAs, lncRNAs are observed to express at a low level, and the expression profile in cancer development is specific [42]. The results also indicated that abundant lncRNAs have differential expression patterns in chordoma. Based on the relationship between lncRNAs and their associated protein-coding genes, we classified the lncRNAs into different groups, including sense, anti-sense, bidirectional, intergenic and intronic lncRNAs [43]. Particularly, there are a large number of intergenic lncRNAs (lincRNA), interspersed in the regions between coding transcripts, found to differentially express in chordomas. lincRNAs were known to exert important functions in various cellular processes, such as maintaining stem cells pluripotency, promoting cell proliferation and accelerating cancer progression [44, 45].

Genomic imprinting is an epigenetic modification of a specific parental chromosome imparted during gametogenesis that induces differential expression of the two alleles [46]. Imprinted genes are known to regulate various physiological processes. Several maternally imprinted genes possess tumor-suppressive potential and were found suppressed in several types of cancer. A large number of lncRNAs, which have been found at all imprinted clusters, are always expressed from the parental chromosome with the unmethylated imprint control region (ICR) [46]. Dysregulation of the lncRNAs in imprinted clusters can silence the imprinted genes, and loss of imprinted genes has been associated with cancer, imprinting-related disease and psychiatric disorders [32]. Comprehensive analysis of lncRNAs and mRNAs expression profiles gives us a better understanding of biological functions of the differentially expressed lncRNAs. Therefore, we performed the correlation analysis of the differentially expressed lncRNAs and protein coding genes, and identified abundant correlations between the transcripts of the two groups. Our lncRNA-coding gene profile revealed concomitant down-regulation of DLK1 and MEG3 in chordoma samples. Maternally expressed gene MEG3 is reciprocally imprinted with the paternally expressed gene DLK1, constituting an imprinting domain on human chromosome 14q32 [47]. MEG3, a large non-coding RNAs as its transcript lacks open reading frame, is positioned about 100 kb from the coding gene DLK1 [47, 48]. MEG3 is involved in various physiological and pathological processes of cell biology, and act as a tumor suppressor through interaction with p53 and MDM2 [32]. MEG3 is highly expressed in normal tissues, including pituitary, brain, adrenal gland and placenta [49]. However, loss of MEG3 expression has been observed in neuroblastomas, hepatocellular cancers and gliomas [50–52]. The inhibition effect of MEG3 on tumor cells proliferation was tested in several cancer cell lines with a number of independent assays, including colony formation, growth curve and BrdU incorporation. Cancer cells with MEG3 overexpression grew significantly slower than the control cells [49, 50]. It is reported that apoptosis induced by MEG3 has also been observed in cancer cells [52]. The collected evidence demonstrated that MEG3 restricts cancer cells biological behavior by inhibiting their proliferation and inducing apoptosis. Further *in vivo* observations confirmed that re-expression of MEG3 in PDFS cells can reduce tumor growth in nude mice [53]. The role of DLK1 in tumor is not clear, and it acts as either an oncogene or tumor suppressor. The DLK1 expression is observed to elevate in gliomas, and overexpression of DKL1 in glioma cell line simulated cells proliferation [54]. In contrast, Kawakami et al. [55] demonstrated that DLK1 expression is lost in human renal cell carcinomas, and its re-expression induced cell death and suppressed tumor growth in nude mice. In several previous reports on other cancer types, either MEG3 or DLK1 was reported to become deregulated, but not both. Under expression of both DLK1 and MEG3 are not always expected, as it is difficult to envision either allelic loss or epitope switching could lead to the concomitant down-regulation of these two imprinted genes. Notably, previous studies also found that miRNAs cluster, which is another major part of DLK1-MEG3 imprinted domain, has also been found to be suppressed or silenced significantly [56, 57]. Therefore, we evaluated the expression of miRNAs in the DLK1-MEG3 locus with microarray, and the results showed that 30 of the 49 miRNAs expression was decreased or lost in chordoma samples, suggesting that silencing may extend across a large part or the entire imprinted gene cluster at 14q32. One of the possible reasons that explain the actual mechanism is novel repressed epigenetic state established during chordoma formation at the 14q32 gene cluster, and this has been demonstrated to occur in urothelial carcinoma [58]. However, further studies should be done to explore the mechanism that inactivates the DLK1-MEG3 imprinting locus in chordoma.

In conclusion, our study mapped the expression profile of lncRNAs and protein coding genes in chordoma, and constructed the lncRNA-coding gene co-expression network. A large number of lncRNAs and protein coding genes expression were found to be dysregulated. *Cis*-regulation analysis and functional assays revealed that the suppression of imprinted gene locus DLK1-MEG3 is crucial to the development in chordoma. Meanwhile, further research work needs to be carried out to explain the function of other lncRNAs and their regulatory effect in chordoma.

## MATERIALS AND METHODS

### Sample preparation and RNA extraction

This study was approved by the Ethical Committee of the First Affiliated Hospital of Soochow University. Written consent was obtained before all subjects were enrolled. The chordoma samples were collected from 3 patients who were identified and treated by tumor resection surgeries at the Department of Orthopedics, the First Affiliated Hospital of Soochow University. The detailed information of the three patients was provided in Supplementary Table 4. FNP specimens were obtained from aborted fetuses with a gestational age of 8–24 weeks in the Department of Gynecology and Obstetrics. As shown in Figure 1, imaging and histopathological analysis confirmed the properties of chordoma and fetal nucleus pulposus tissues. Following informed consent at the time of acquisition, the samples were collected and stored in liquid nitrogen. Tissue samples were homogenized in TRIzol reagent (Ambion). In our study, 3 chordoma samples and 3 FNP samples were subjected to the microarray analysis. Total RNA was quantified by the NanoDrop ND-2000 (Thermo Scientific), and the RNA integrity was assessed using Agilent Bioanalyzer 2100 (Agilent Technologies).

### Microarray assays

The chordoma and fetal nucleus pulposus samples RNAs were employed to synthesize double-stranded complementary DNA (cDNA), and then labeled with the Agilent Human lncRNA (4^*^180K, Design ID:062918) and Agilent Human miRNA (8^*^60K, Design ID: 046064). Microarray hybridization and washing were performed based on the manufacturer's standard protocols. Briefly, total RNA were transcribed to double strand cDNA, then synthesized into cRNA and labeled with Cyanine-3-CTP. The labeled cDNAs were hybridized onto the microarray. After washing, the arrays were scanned by the Agilent Scanner G2505C (Agilent Technologies).

### Microarray data analysis

Feature Extraction software (version10.7.1.1, Agilent Technologies) was used to analyze array images in order to get the raw data. Genespring was employed to perform the basic analysis with the raw data. Firstly, the raw data was normalized with the quantile algorithm. Differentially expressed genes or lncRNAs were then identified through fold change as well as *P* value assessed by student *t*-test. The threshold set for differentially expressed genes were fold change more than 2.0, and the *P* value less than 0.05. Afterward, Hierarchical Clustering was performed to display the distinguishable genes’ expression pattern among samples. Treeview software (Stanford university, CA, USA) composed by Java was utilized to visualize the microarray data.

### qPCR analysis

The total RNA was extracted from the chordoma samples and FNP tissues collected for microarray assay by using TRIzol reagent (Invitrogen, CA,USA). Then, the extracted RNA was reversely transcribed using PrimeScript RT reagent kit with gDNA Eraser (Perfect Real Time) (TaKaRa, Dalian, China) according to the manufacture's recommendations. The primers sequences for the selected lncRNAs and protein coding genes were listed in Supplementary Table 5. The real-time PCR was performed by using SYBRGreen (TaKaRa, Dalian, China), and GAPDH was employed as the internal control. The expression of each lncRNA was represented as fold change using 2^-ΔΔCt^ [59]. Student *t*-test was utilized to analyze the significance of the expression between chordoma samples and their control samples. The value of *P* < 0.05 was considered significant.

### Correlation analysis between lncRNA and mRNA

The network between lncRNA and mRNA was constructed based on the correlation analysis among differentially expressed lncRNAs and protein coding genes. For each pair, Pearson correlation was performed to assess the correlation. For the pairs with absolute value of Pearson correlation coefficients not less than 0.90 were selected to draw the network using Cytoscape (National Resource for Network Biology).

### KEGG pathway analysis

To identify the pathways of intersect, we performed Kyoto Encyclopedia of Genes and Genomes (KEGG, http://www.genome.ad.jp/kegg/) enrichment analysis. The Fisher's exact test, χ^2^ test and the threshold of significance were conducted for the analysis. The *P* value and FDR were calculated, and *P* < 0.05 was used as the screening criteria.

### *Cis*-correlation analysis

The cis-regulation regions were identified by the following procedures. For each lncRNAs, we identified the mRNAs as “cis-regulated mRNAs” when: (1) the mRNAs locus are within 300k windows up- and downstream of the given lncRNA, (2) the Pearson correlation of lncRNA-mRNA expression is significant (*P*-value of correlation less than 0.05).

### Cell culture and transfection

The human chordoma cell line U-CH1 was obtained from ATCC (USA). Chordoma cells were cultured in IMDM and RPMI1640 (1:4) supplemented with 10% fetal bovine serum (FBS), and penicillin/streptomycin (100 U/ml) (Invitrogen, CA,USA). The cells were cultured in an incubator at 37°C and an atmosphere of 5% CO_2_, and the medium was changed every 3 days. The cells were sub-cultured once in confluence. The MEG3 overexpression vector (pcDNA-MEG3) and the corresponding control (empty pcDNA) were constructed. MEG3 sequence was synthesized and subcloned into pcDNA3.1. The gene of lncRNA MEG3 was confirmed by restriction enzyme digestion (Supplementary Figure 1). The constructed pcDNA-MEG3 and the empty pcDNA were respectively transfected into chordoma cells using Lipofectamine 2000 reagent (Invitrogen, CA,USA). RNA extraction and EdU staining assay were performed 3 days after transfection.

### Cell cycle analysis

For cell cycle analysis, the chordoma cells were fixed with ice-cold 70% ethanol for 12 hours at −20°C. Then the cells were washed and re-suspended and stained with propidium iodide solution (Invitrogen, CA,USA) at the concentration of 5ug/ml. The cells were analyzed by Flow Cytometry (BD Biosciences, Franklin Lakes, NJ, USA).

### EdU staining assay

EdU staining for chordoma cell proliferation was assessed using the Cell-Light™ EdU detection kit (RiboBio, Guangzhou, China) according to the manufacturer's instructions. The chordoma cells were incubated with 10 μM EdU for 2 hours before fixed with 4% paraformaldehyde. After fixation, the chordoma cells were permeabilizated using 0.5% Triton X-100 for 10 min. Then, the cells were stained with Apollo643 for 30 min, and subsequently stained with Hoechst. Fluorescent cells were counted using confocal microscope.

### Statistical analysis

Statistical analysis was performed with SPSS 17.0 software. Student *t*-test was used to compare the significance between control and MEG3 overexpression groups. All the data were expressed as mean ± standard deviation (SD). *P* value < 0.05 was considered as significant, and *P* value < 0.01 was considered as highly significant.

## SUPPLEMENTARY MATERIALS FIGURE AND TABLES









## Abbreviations

RTKs: Receptor tyrosine kinases
EGFR: epidermal growth factor receptor
miRNAs: microRNAs
lncRNAs: long noncoding RNAs
PRC2: polycomb repressive, complex2
FNP: fetal nucleus pulposus
CT: computed tomography
MRI: magnetic resonance imaging
FC: fold change
PDGFR: platelet-derived growth factor receptor
IGF-1R: Insulin-like Growth Factor-1R
lincRNA: intergenic lncRNAs
MEG3: maternal expressed gene 3
cDNA: complementary DNA
qPCR: quantitative polymerase chain reaction

## References

[1] Gulluoglu S, Turksoy O, Kuskucu A, Ture U, Bayrak OF (2016). The molecular aspects of chordoma. Neurosurg Rev.

[2] Bergh P, Kindblom LG, Gunterberg B, Remotti F, Ryd W, Meis-Kindblom JM (2000). Prognostic factors in chordoma of the sacrum and mobile spine: a study of 39 patients. Cancer.

[3] Schwab JH, Boland PJ, Agaram NP, Socci ND, Guo T, O’Toole GC, Wang X, Ostroumov E, Hunter CJ, Block JA, Doty S, Ferrone S, Healey JH (2009). Chordoma and chondrosarcoma gene profile: implications for immunotherapy. Cancer Immunol Immunother.

[4] Walcott BP, Nahed BV, Mohyeldin A, Coumans JV, Kahle KT, Ferreira MJ (2012). Chordoma: current concepts, management, and future directions. Lancet Oncol.

[5] Shalaby A, Presneau N, Ye H, Halai D, Berisha F, Idowu B, Leithner A, Liegl B, Briggs TR, Bacsi K, Kindblom LG, Athanasou N, Amary MF (2011). The role of epidermal growth factor receptor in chordoma pathogenesis: a potential therapeutic target. J Pathol.

[6] Yu X, Li Z (2015). Epigenetic deregulations in chordoma. Cell Prolif.

[7] Lee JJ, Murphy GF, Lian CG (2014). Melanoma epigenetics: novel mechanisms, markers, and medicines. Lab Invest.

[8] Deans C, Maggert KA (2015). What do you mean, “epigenetic”?. Genetics.

[9] Feinberg AP, Tycko B (2004). The history of cancer epigenetics. Nat Rev Cancer.

[10] Bayrak OF, Gulluoglu S, Aydemir E, Ture U, Acar H, Atalay B, Demir Z, Sevli S, Creighton CJ, Ittmann M, Sahin F, Ozen M (2013). MicroRNA expression profiling reveals the potential function of microRNA-31 in chordomas. J Neurooncol.

[11] Duan Z, Choy E, Nielsen GP, Rosenberg A, Iafrate J, Yang C, Schwab J, Mankin H, Xavier R, Hornicek FJ (2010). Differential expression of microRNA (miRNA) in chordoma reveals a role for miRNA-1 in Met expression. J Orthop Res.

[12] Duan Z, Shen J, Yang X, Yang P, Osaka E, Choy E, Cote G, Harmon D, Zhang Y, Nielsen GP, Spentzos D, Mankin H, Hornicek F (2014). Prognostic significance of miRNA-1 (miR-1) expression in patients with chordoma. J Orthop Res.

[13] Iacoangeli A, Lin Y, Morley EJ, Muslimov IA, Bianchi R, Reilly J, Weedon J, Diallo R, Bocker W, Tiedge H (2004). BC200 RNA in invasive and preinvasive breast cancer. Carcinogenesis.

[14] Yuan JH, Yang F, Wang F, Ma JZ, Guo YJ, Tao QF, Liu F, Pan W, Wang TT, Zhou CC, Wang SB, Wang YZ, Yang Y (2014). A long noncoding RNA activated by TGF-beta promotes the invasion-metastasis cascade in hepatocellular carcinoma. Cancer Cell.

[15] Svoboda M, Slyskova J, Schneiderova M, Makovicky P, Bielik L, Levy M, Lipska L, Hemmelova B, Kala Z, Protivankova M, Vycital O, Liska V, Schwarzova L (2014). HOTAIR long non-coding RNA is a negative prognostic factor not only in primary tumors, but also in the blood of colorectal cancer patients. Carcinogenesis.

[16] Ren S, Wang F, Shen J, Sun Y, Xu W, Lu J, Wei M, Xu C, Wu C, Zhang Z, Gao X, Liu Z, Hou J (2013). Long non-coding RNA metastasis associated in lung adenocarcinoma transcript 1 derived miniRNA as a novel plasma-based biomarker for diagnosing prostate cancer. Eur J Cancer.

[17] Fatica A, Bozzoni I (2014). Long non-coding RNAs: new players in cell differentiation and development. Nat Rev Genet.

[18] Wang X, Arai S, Song X, Reichart D, Du K, Pascual G, Tempst P, Rosenfeld MG, Glass CK, Kurokawa R (2008). Induced ncRNAs allosterically modify RNA-binding proteins in cis to inhibit transcription. Nature.

[19] Lin D, Pestova TV, Hellen CU, Tiedge H (2008). Translational control by a small RNA: dendritic BC1 RNA targets the eukaryotic initiation factor 4A helicase mechanism. Mol Cell Biol.

[20] Beltran M, Puig I, Pena C, Garcia JM, Alvarez AB, Pena R, Bonilla F, de Herreros AG (2008). A natural antisense transcript regulates Zeb2/Sip1 gene expression during Snail1-induced epithelial-mesenchymal transition. Genes Dev.

[21] Khalil AM, Guttman M, Huarte M, Garber M, Raj A, Rivea Morales D, Thomas K, Presser A, Bernstein BE, van Oudenaarden A, Regev A, Lander ES, Rinn JL (2009). Many human large intergenic noncoding RNAs associate with chromatin-modifying complexes and affect gene expression. Proc Natl Acad Sci USA.

[22] Mattick JS (2009). The genetic signatures of noncoding RNAs. PLoS Genet.

[23] Chu C, Qu K, Zhong FL, Artandi SE, Chang HY (2011). Genomic maps of long noncoding RNA occupancy reveal principles of RNA-chromatin interactions. Mol Cell.

[24] Gabory A, Jammes H, Dandolo L (2010). The H19 locus: role of an imprinted non-coding RNA in growth and development. Bioessays.

[25] Pauler FM, Koerner MV, Barlow DP (2007). Silencing by imprinted noncoding RNAs: is transcription the answer?. Trends Genet.

[26] Mancini-Dinardo D, Steele SJ, Levorse JM, Ingram RS, Tilghman SM (2006). Elongation of the Kcnq1ot1 transcript is required for genomic imprinting of neighboring genes. Genes Dev.

[27] Li Y, Sasaki H (2011). Genomic imprinting in mammals: its life cycle, molecular mechanisms and reprogramming. Cell Res.

[28] Mohammad F, Mondal T, Kanduri C (2009). Epigenetics of imprinted long noncoding RNAs. Epigenetics.

[29] Nagano T, Mitchell JA, Sanz LA, Pauler FM, Ferguson-Smith AC, Feil R, Fraser P (2008). The Air noncoding RNA epigenetically silences transcription by targeting G9a to chromatin. Science.

[30] Zhang X, Gejman R, Mahta A, Zhong Y, Rice KA, Zhou Y, Cheunsuchon P, Louis DN, Klibanski A (2010). Maternally expressed gene 3, an imprinted noncoding RNA gene, is associated with meningioma pathogenesis and progression. Cancer Res.

[31] Bartolomei MS, Ferguson-Smith AC (2011). Mammalian genomic imprinting. Cold Spring Harb Perspect Biol.

[32] Benetatos L, Vartholomatos G, Hatzimichael E (2011). MEG3 imprinted gene contribution in tumorigenesis. Int J Cancer.

[33] Ponting CP, Oliver PL, Reik W (2009). Evolution and functions of long noncoding RNAs. Cell.

[34] Tsai MC, Spitale RC, Chang HY (2011). Long intergenic noncoding RNAs: new links in cancer progression. Cancer Res.

[35] de Castro CV, Guimaraes G, Aguiar S, Lopes A, Baiocchi G, da Cunha IW, Campos AH, Soares FA, Begnami MD (2013). Tyrosine kinase receptor expression in chordomas: phosphorylated AKT correlates inversely with outcome. Hum Pathol.

[36] Tamborini E, Virdis E, Negri T, Orsenigo M, Brich S, Conca E, Gronchi A, Stacchiotti S, Manenti G, Casali PG, Pierotti MA, Pilotti S (2010). Analysis of receptor tyrosine kinases (RTKs) and downstream pathways in chordomas. Neuro Oncol.

[37] Tamborini E, Miselli F, Negri T, Lagonigro MS, Staurengo S, Dagrada GP, Stacchiotti S, Pastore E, Gronchi A, Perrone F, Carbone A, Pierotti MA, Casali PG (2006). Molecular and biochemical analyses of platelet-derived growth factor receptor (PDGFR) B, PDGFRA, and KIT receptors in chordomas. Clin Cancer Res.

[38] Scheipl S, Froehlich EV, Leithner A, Beham A, Quehenberger F, Mokry M, Stammberger H, Varga PP, Lazary A, Windhager R, Gattenloehner S, Liegl B (2012). Does insulin-like growth factor 1 receptor (IGF-1R) targeting provide new treatment options for chordomas? A retrospective clinical and immunohistochemical study. Histopathology.

[39] Sommer J, Itani DM, Homlar KC, Keedy VL, Halpern JL, Holt GE, Schwartz HS, Coffin CM, Kelley MJ, Cates JM (2010). Methylthioadenosine phosphorylase and activated insulin-like growth factor-1 receptor/insulin receptor: potential therapeutic targets in chordoma. J Pathol.

[40] Grabellus F, Konik MJ, Worm K, Sheu SY, van de Nes JA, Bauer S, Paulus W, Egensperger R, Schmid KW (2010). MET overexpressing chordomas frequently exhibit polysomy of chromosome 7 but no MET activation through sarcoma-specific gene fusions. Tumour Biol.

[41] Naka T, Iwamoto Y, Shinohara N, Ushijima M, Chuman H, Tsuneyoshi M (1997). Expression of c-met proto-oncogene product (c-MET) in benign and malignant bone tumors. Mod Pathol.

[42] Derrien T, Johnson R, Bussotti G, Tanzer A, Djebali S, Tilgner H, Guernec G, Martin D, Merkel A, Knowles DG, Lagarde J, Veeravalli L, Ruan X (2012). The GENCODE v7 catalog of human long noncoding RNAs: analysis of their gene structure, evolution, and expression. Genome Res.

[43] Kung JT, Colognori D, Lee JT (2013). Long noncoding RNAs: past, present, and future. Genetics.

[44] Huarte M, Guttman M, Feldser D, Garber M, Koziol MJ, Kenzelmann-Broz D, Khalil AM, Zuk O, Amit I, Rabani M, Attardi LD, Regev A, Lander ES (2010). A large intergenic noncoding RNA induced by p53 mediates global gene repression in the p53 response. Cell.

[45] Prensner JR, Iyer MK, Balbin OA, Dhanasekaran SM, Cao Q, Brenner JC, Laxman B, Asangani IA, Grasso CS, Kominsky HD, Cao X, Jing X, Wang X (2011). Transcriptome sequencing across a prostate cancer cohort identifies PCAT-1, an unannotated lincRNA implicated in disease progression. Nat Biotechnol.

[46] Benetatos L, Voulgaris E, Vartholomatos G (2012). DLK1-MEG3 imprinted domain microRNAs in cancer biology. Crit Rev Eukaryot Gene Expr.

[47] Schmidt JV, Matteson PG, Jones BK, Guan XJ, Tilghman SM (2000). The Dlk1 and Gtl2 genes are linked and reciprocally imprinted. Genes Dev.

[48] Wylie AA, Murphy SK, Orton TC, Jirtle RL (2000). Novel imprinted DLK1/GTL2 domain on human chromosome 14 contains motifs that mimic those implicated in IGF2/H19 regulation. Genome Res.

[49] Zhang X, Zhou Y, Mehta KR, Danila DC, Scolavino S, Johnson SR, Klibanski A (2003). A pituitary-derived MEG3 isoform functions as a growth suppressor in tumor cells. J Clin Endocrinol Metab.

[50] Braconi C, Kogure T, Valeri N, Huang N, Nuovo G, Costinean S, Negrini M, Miotto E, Croce CM, Patel T (2011). microRNA-29 can regulate expression of the long non-coding RNA gene MEG3 in hepatocellular cancer. Oncogene.

[51] Astuti D, Latif F, Wagner K, Gentle D, Cooper WN, Catchpoole D, Grundy R, Ferguson-Smith AC, Maher ER (2005). Epigenetic alteration at the DLK1-GTL2 imprinted domain in human neoplasia: analysis of neuroblastoma, phaeochromocytoma and Wilms’ tumour. Br J Cancer.

[52] Wang P, Ren Z, Sun P (2012). Overexpression of the long non-coding RNA MEG3 impairs *in vitro* glioma cell proliferation. J Cell Biochem.

[53] Zhou Y, Zhang X, Klibanski A (2012). MEG3 noncoding RNA: a tumor suppressor. J Mol Endocrinol.

[54] Yin D, Xie D, Sakajiri S, Miller CW, Zhu H, Popoviciu ML, Said JW, Black KL, Koeffler HP (2006). DLK1: increased expression in gliomas and associated with oncogenic activities. Oncogene.

[55] Kawakami T, Chano T, Minami K, Okabe H, Okada Y, Okamoto K (2006). Imprinted DLK1 is a putative tumor suppressor gene and inactivated by epimutation at the region upstream of GTL2 in human renal cell carcinoma. Hum Mol Genet.

[56] Qin R, Chen Z, Ding Y, Hao J, Hu J, Guo F (2013). Long non-coding RNA MEG3 inhibits the proliferation of cervical carcinoma cells through the induction of cell cycle arrest and apoptosis. Neoplasma.

[57] Ying L, Huang Y, Chen H, Wang Y, Xia L, Chen Y, Liu Y, Qiu F (2013). Downregulated MEG3 activates autophagy and increases cell proliferation in bladder cancer. Mol Biosyst.

[58] Greife A, Knievel J, Ribarska T, Niegisch G, Schulz WA (2014). Concomitant downregulation of the imprinted genes DLK1 and MEG3 at 14q32.2 by epigenetic mechanisms in urothelial carcinoma. Clin Epigenetics.

[59] Pfaffl MW (2001). A new mathematical model for relative quantification in real-time RT-PCR. Nucleic Acids Res.

