# Supplementary material for: Comprehensive analysis of mRNA-lncRNA co-expression profile revealing crucial role of imprinted gene cluster DLK1-MEG3 in chordoma
Source: Oncotarget. 2017 Nov 8;8(68):112623–35. doi: 10.18632/oncotarget.22616 (PMC5762536; doi:10.18632/oncotarget.22616)
Supplement: Supplementary file 1 [file oncotarget-08-112623-s001.pdf]

Comprehensive analysis of mRNA-lncRNA co-expression profile revealing crucial role of imprinted gene cluster DLK1-MEG3 in chordoma

SUPPLEMENTARY MATERIALS

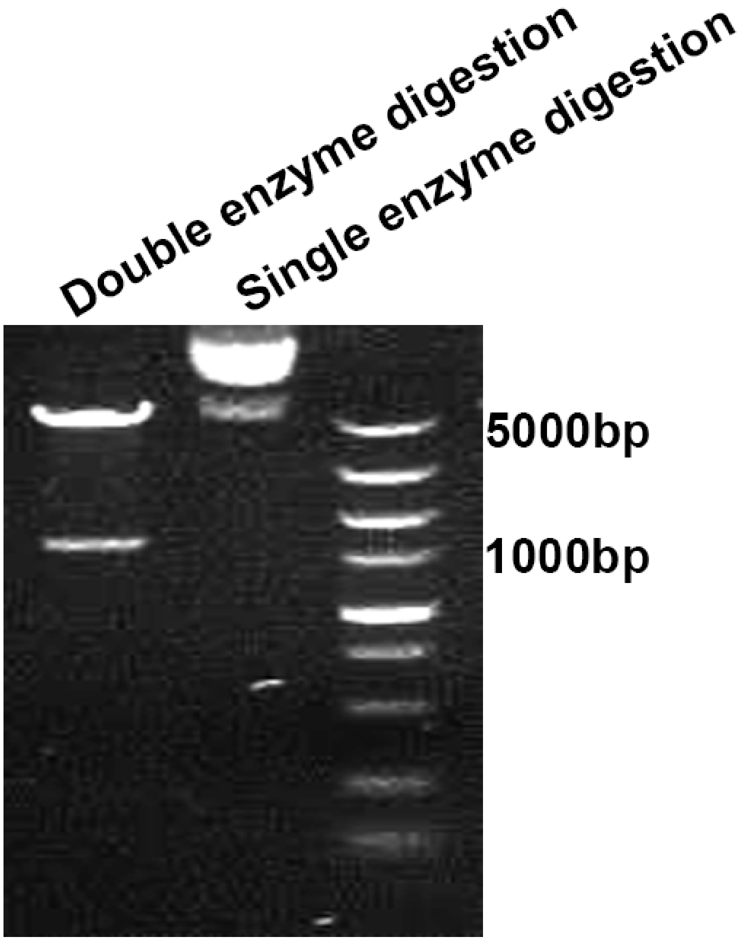

Supplementary Figure 1: Identification of the MEG3 overexpression vector(pcDNA-MEG3) with double and single enzyme digestion.

Supplementary Table 1: Diferentially expressed mRNAs. See Supplementary_Table_1

Supplementary Table 2: Differentially expressed lncRNAs. See Supplementary_Table_2

Supplementary Table 3: lncRNA-mRNA corelation. See Supplementary_Table_3

Supplementary Table 4: The demographic information of the chordoma patients

	**Case1**	**Case2**	**Case3**
Gender	Female	Male	Male
Age	33	58	83
Location	S4	S4-5	S3
Other diseases	No	No	No

Supplementary Table 5: Primers for qPCR

**Gene name**	**Forward**	**Reverse**
IGF1R	ATGCTTCAGAAACACCTCAAT	AGGCTAGAACTGTGGATAGAT
EGFR	GAGTATATGTTCCCTCCAGGT	AGAGCTGTAAGTGCTTGAATAG
MET	TCCAATCACAGCTCATAGGTAG	TTTGTTTCCAATTGCTTGCGA
PDGFRB	CATAGCAAGTGCCTGTGT	GGCCTTCTAGACTCTGGT
DLK1	GAGAGGAGGAAGAGGAGTATCA	GCATGTCTGGACTGGTAGTAA
NR_033359.1	CGTCTCCTTCCTGGTTTG	TGGCTGTGGAGGGATTTC
NONHSAT054600	GCCTATCACTTCAAGCGT	TGACCTTGAGCTTGGACT
ENST00000553465	CAACTCAGGTCTGTGTGATTC	CCAGCCATCTCTCCATCTAT
NONHSAT097304	GGGATTACAGACTACAAAGGC	CAGCAGCAAACTTGCTTGA
MEG3	GCAGGATCTGGCATAGAGGA	CCTGGAGTGCTGTTGGAGA
P53	GCACTGTCCAACAACACCAG	CCTCATTCAGCTCTCGGAAC
Bcl2	GGAGGATTGTGGCCTTCTT	GCCGGTTCAGGTACTCAGTC
GAPDH	TGTTGCCATCAATGACCCCTT	CTCCACGACGTACTCAGCG
